# AMAnD: an automated metagenome anomaly detection methodology utilizing DeepSVDD neural networks

**DOI:** 10.3389/fpubh.2023.1181911

**Published:** 2023-07-11

**Authors:** Colin Price, Joseph A. Russell

**Affiliations:** Life Science Resource Center, MRIGlobal, Gaithersburg, MD, United States

**Keywords:** anomaly detection, metagenomics, deep learning, DeepSVDD, machine learning

## Abstract

The composition of metagenomic communities within the human body often reflects localized medical conditions such as upper respiratory diseases and gastrointestinal diseases. Fast and accurate computational tools to flag anomalous metagenomic samples from typical samples are desirable to understand different phenotypes, especially in contexts where repeated, long-duration temporal sampling is done. Here, we present Automated Metagenome Anomaly Detection (AMAnD), which utilizes two types of Deep Support Vector Data Description (DeepSVDD) models; one trained on taxonomic feature space output by the Pan-Genomics for Infectious Agents (PanGIA) taxonomy classifier and one trained on kmer frequency counts. AMAnD's semi-supervised one-class approach makes no assumptions about what an anomaly may look like, allowing the flagging of potentially novel anomaly types. Three diverse datasets are profiled. The first dataset is hosted on the National Center for Biotechnology Information's (NCBI) Sequence Read Archive (SRA) and contains nasopharyngeal swabs from healthy and COVID-19-positive patients. The second dataset is also hosted on SRA and contains gut microbiome samples from normal controls and from patients with slow transit constipation (STC). AMAnD can learn a typical healthy nasopharyngeal or gut microbiome profile and reliably flag the anomalous COVID+ or STC samples in both feature spaces. The final dataset is a synthetic metagenome created by the Critical Assessment of Metagenome Annotation Simulator (CAMISIM). A control dataset of 50 well-characterized organisms was submitted to CAMISIM to generate 100 synthetic control class samples. The experimental conditions included 12 different spiked-in contaminants that are taxonomically similar to organisms present in the laboratory blank sample ranging from one strain tree branch taxonomic distance away to one family tree branch taxonomic distance away. This experiment was repeated in triplicate at three different coverage levels to probe the dependence on sample coverage. AMAnD was again able to flag the contaminant inserts as anomalous. AMAnD's assumption-free flagging of metagenomic anomalies, the real-time model training update potential of the deep learning approach, and the strong performance even with lightweight models of low sample cardinality would make AMAnD well-suited to a wide array of applied metagenomics biosurveillance use-cases, from environmental to clinical utility.

## 1. Introduction

The applicability of regular sequencing applications that monitor compositions of soil (1), wastewater (2), built-environment (3), clinical (4, 5), and other types of microbiomes are increasingly salient as climate change and synthetic biology technologies complicate the infectious disease and biothreat landscape (6, 7). Research and development of shotgun metagenomics biosurveillance protocols have led to an exponentially increasing amount of publicly available metagenomic data (8). A common prompt of metagenomics inquiry (particularly in routine monitoring applications) is whether there is genetic material in the sample that is not expected to be present.

Anomalies can take many different forms depending on context. In a lab environment, technician errors may introduce human materials or other contaminants (9). Changes in environmental factors may make a soil environment favorable for a new ecologically disruptive microbe or genotype (10). A human infected with a pathogen or suffering an adverse clinical condition may experience shifts in the species distribution of their microbiome (11). In some cases, the context of what an anomalous metagenomic sample looks like can be known *a priori*, but in more dynamic or less-routinely sampled environments (e.g., the cabin and crew of a deep space flight), little is known of how a microbiome might change, yet these shifts may indicate a need for immediate remedial action. Across metagenomics, there is a need for computational metagenomic anomaly detection that works with high fidelity and can detect a broad spectrum of possible anomalies while making few assumptions about what form an anomaly might take.

The current state-of-the-art methods for metagenomic signal detection include a variety of machine learning-based methods including clustering-based methods like k-means, linear models like Support Vector Machine, and non-linear tree or neural network-based models (12–14). The increasing availability of high-dimensional metagenomics data lends itself well to machine learning methods that can glean complex insights into the problem in any given domain. Specifically, for anomaly detection in metagenomics applications, the “Squeegee” algorithm was developed to identify and remove contaminating sequences from applied metagenomics datasets (15). Squeegee accomplishes this by matching k-mers—short sequences of DNA of length *k*—to identified contaminant candidate organisms and removing sequences matching these k-mer profiles from the analysis. While this approach has been shown to be effective at improving the resolution of “on-target” signals of interest, it still requires *a priori* knowledge of what anomalous metagenomic signatures may look like.

Here, we describe AMAnD—automated metagenome anomaly detection—methodology utilizing DeepSVDD neural networks. AMAnD is designed to identify and flag anomalous metagenomic samples that diverge from standard or “normal” signatures produced through routine metagenomic monitoring. AMAnD leverages two metagenomics signature types—k-mers and taxonomic classifications—to identify anomalies but requires no *a priori* definition of what a potential anomaly may present as. AMAnD is trained on a historical corpus of representative samples from user-defined standard conditions and, when presented with a new dataset, will decide whether the new sample is representative of historical trends. By leveraging the DeepSVDD architecture and two feature spaces (k-mers and taxonomies), AMAnD is flexible and can capture more complex patterns in metagenomics data than other metagenomics-anomaly/contaminant-detection algorithms. By not requiring pre-definition of problematic or contaminating sequence types, it can more reliably flag samples that may be anomalous in ways a user does not expect. This is especially useful for emerging paradigms where deployed metagenomics biosurveillance can add a lot of value, including wastewater surveillance, agricultural or zoonotic reservoir surveillance, and crew health monitoring in extreme environments (e.g., long-duration, human-occupied spacecraft).

## 2. Methods

### 2.1. Selection of DeepSVDD as methodology

One-class classification is well-suited to problems where the dataset is imbalanced or if one class is unavailable at training time (16). Anomaly detection is an example of a highly imbalanced data problem—e.g., the majority of the data passing through the network is of the typical “normal” class, which makes one-class detection a strong approach. Another advantage of one-class classification is that there are no assumptions imparted at training time about the nature of the anomaly. Two-class classification models make labeling decisions (for example, normal or anomalous) by learning both the typical control profile as well as an anomalous profile. The learning of an anomalous profile assumes that future anomalies will all be similar to those used at training time which may limit the ability to flag samples that are anomalous in unexpected ways.

Autoencoders and variational autoencoders are popular choices for anomaly detection across many fields of application (17, 18). Autoencoder-like networks compress the input signal through multiple, successively smaller, feed-forward layers of activation functions to learn weights that compress the signal into a representative low-level feature space. A mirrored network on the opposite side then learns weights to expand and reconstruct the low-level feature space back into the original signal. A threshold or tolerance between the difference of the original signal and the reconstructed signal (also known as “anomaly score” or “reconstruction score”) is learned based on the variance between the inputs such that any difference too great between the original signal and reconstructed signal is flagged as anomalous.

DeepSVDD expands on the autoencoder-like network approach to anomaly detection by learning a function that describes a minimal *n*-dimensional hypersphere that encloses the representation of the typical normal class (19, 20). This means defining a center and the smallest radius that encloses the maximum data within any given feature space (19). The outer edge of the hypersphere can be thought of as a threshold; data points outside the hypersphere radius are labeled as anomalous and data points inside are considered normal. DeepSVDD was designed for anomaly detection use-cases and naturally can be used to extend the advantages of autoencoder with the ability to learn the *n*-dimensional hypersphere on the compressed feature space of the autoencoder network. AMAnD uses the PyOD library's autoencoder, based on *tensorflow*, which has been benchmarked well against other anomaly detectors (21). The mathematics behind the DeepSVDD model is detailed more in Supplementary Figure 1.

### 2.2. Selection of feature vectors

AMAnD was profiled on two feature spaces chosen to cover, in ensemble, the diversity of possible anomalies that may be present in a metagenomic dataset. The first feature vector is the output of the PanGIA (22) taxonomy classifier which is filtered down to attributions of reads at the genus, species, and strain levels crossed with two different read count normalizations including; reads normalized to reference (RNR) and reads normalized by sequence identity (RNI). RNR is the value of the number of reads mapping to a given reference divided by the total number of other references that each of those reads map to at the defined mapping stringency. RNI is the sum of all reads—each read normalized by its percent-identity to the region it maps to—mapping to a given reference. The RNR and RNI up-weight the magnitudes of reads mapping uniquely to a reference and are available for each taxonomy identified in the sample. This yields six descriptive metrics (strain RNR, strain RNI, species RNR, species RNI, genus RNR, and genus RNI) for each taxonomy identified in a sample. Different truncations of the total number of metrics included in the feature vector are searched over at training time, keeping the top *n* highest-magnitude values from the PanGIA report. The top *n* highest-magnitude values are kept in the order output by the PanGIA report, which implicitly captures the taxonomic classification information. This feature vector should capture anomalies that are comprised of the presence of a small number of anomalous taxa or the absence of a small number of expected taxa. PanGIA will always assign a label and will assign it to whatever the closest taxa present in the PanGIA database. This implicitly captures the taxonomy regardless of if it is labeled properly, as the DeepSVDD portion of AMAnD does not explicitly check for the presence/absence of a taxon.

The second feature vector chosen is k-mer counts. The Jellyfish k-mer counter (23) was used to profile 3mers or 4mers which have the counts processed into AMAnD. Small k-mers can characterize community compositions similarly to taxonomies but can capture shifts in communities that may not be reflected by taxonomic labels (12), i.e., a metagenomic community that remains taxonomically consistent may have genotype shifts that functionally change the community that is reflected in k-mer frequency. 3mers and 4mers are chosen to keep the total size of the feature vector small, as the cardinality of k-mers grows exponentially and larger k-mers would require some level of feature selection to keep the feature vector size small enough to avoid complications of high dimensionality.

### 2.3. The AMAnD pipeline

**Figure 1:**
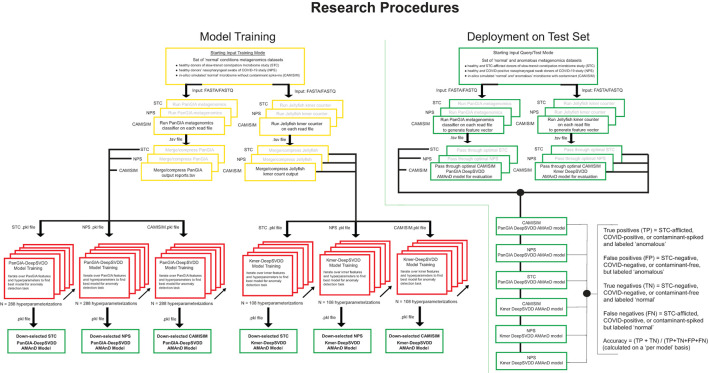
Overview of model development, training, and validation of the AMAnD method. Models were developed for selected publicly available microbiome datasets associated with two human health conditions; slow-transit constipation (STC) and COVID-19 nasopharyngeal swabs (NPS). In addition, *in silico* simulated metagenomic samples (across 3 simulated coverage levels) were manipulated with sequences from anomalous spike-in organisms at increasing taxonomic divergence from community members in the “normal” condition samples using the tool suite from CAMISIM.

For training, the input to the AMAnD pipeline was a set of “standard conditions” metagenomic short read files in fasta or fastq format. Reads were passed to the PanGIA taxonomy classifier and Jellyfish k-mer counter in parallel. Taxonomy reports generated by PanGIA and Jellyfish 3mers and 4mers were then merged into feature vectors that are passed on to DeepSVDD from the PyOD library. A grid search is then performed across 288 parameterizations of PanGIA feature vector models and 108 parameterizations of k-mer feature vector models. The sets of hyperparameters searched over include different hidden layer dimensions, feature vector truncation length, drop-out rate, L2 regularization coefficient, and internal autoencoder use (Figure 1; Supplementary Figure 2; Supplementary Table 1). Each resultant model has learned a minimal n-dimensional hypersphere, which is used to accept or reject input samples akin to a threshold.

A 10% holdout validation set is used to assess the viability of the model, and the parameterization that minimizes the difference in the mean squared error of reconstruction between the training and validation sets is used as an initial down-selection of model hyperparameters. In this screening, the models with the minimal validation error are captured and only models with decreasing and minimal error (per visual screening) are evaluated against the test set to choose the optimal model. This was performed by holistic human interpretation of the entire training plot over the 400 epochs. Examples of down-selected models' training behavior with minimal training and validation error are shown in Supplementary Figure 3. Since both sets are members of the same normal class, observing the reconstruction MSE over the training epochs of both training and validation suggests that the model generalizes to unseen data well. Unseen validation control data points must be reconstructed as well as the training in the anomaly detection case to avoid false positive flagging of anomalies. The best models presented in this study are the models that perform with the highest accuracy of classification on both the validation holdout normal class set and the test anomaly set which is not used in training. With the optimal model chosen, future single samples can be passed for anomaly screening. The metagenomic sample is passed through PanGIA and Jellyfish to generate feature taxonomy and k-mer vectors that are then passed to the optimal model scoring for assessment (Figure 1).

### 2.4. AMAnD profiling

AMAnD was profiled for performance on three distinct datasets—two datasets collected from the National Center for Biotechnology Information's (NCBI) Sequence Read Archive (SRA) and one synthetic dataset generated by the Critical Assessment of Metagenome Interpretation simulator (CAMISIM) (24). The first dataset was collected from human nasopharyngeal microbiome swabs (NPS) from COVID-19-positive and COVID-19-negative patients (11). Feehan et al. found differences in metagenomic community abundances of *Serratia* spp., *Streptococcus* spp., *Enterobacter* spp., *Veillonella* spp., *Prevotella* spp., *and Rothia* spp. in COVID-19-positive samples. Control COVID-19-negative samples from healthy donors of the study were used to train an AMAnD model as the typical metagenomic background profile and COVID-19-positive samples were used as an anomaly test set. Samples were chunked into files of 1,000,000 reads each to bolster sample numbers, resulting in a dataset of size *n* = 988. The second dataset was collected from human gut microbiome samples from patients with slow transit constipation (STC) and healthy control patients (25). Tian et al. found Shannon and Simpson diversity metrics to be significantly higher in the STC group than in healthy controls. Healthy control samples were used to train an AMAnD model as the typical background model and the STC samples were used as an anomaly test set. Samples were again chunked into files of 1,000,000 reads to bolster sample numbers, resulting in a dataset size of *n* = 724. The datasets were chosen because the publications associated with them had independently validated there were substantial differences between the two groups which should be reliably detectable. Furthermore, validation of these datasets also suggests that AMAnD is appropriate for monitoring nuanced shifts in human metagenomic samples that may serve as early indicators of deleterious health conditions.

**Figure 2:**
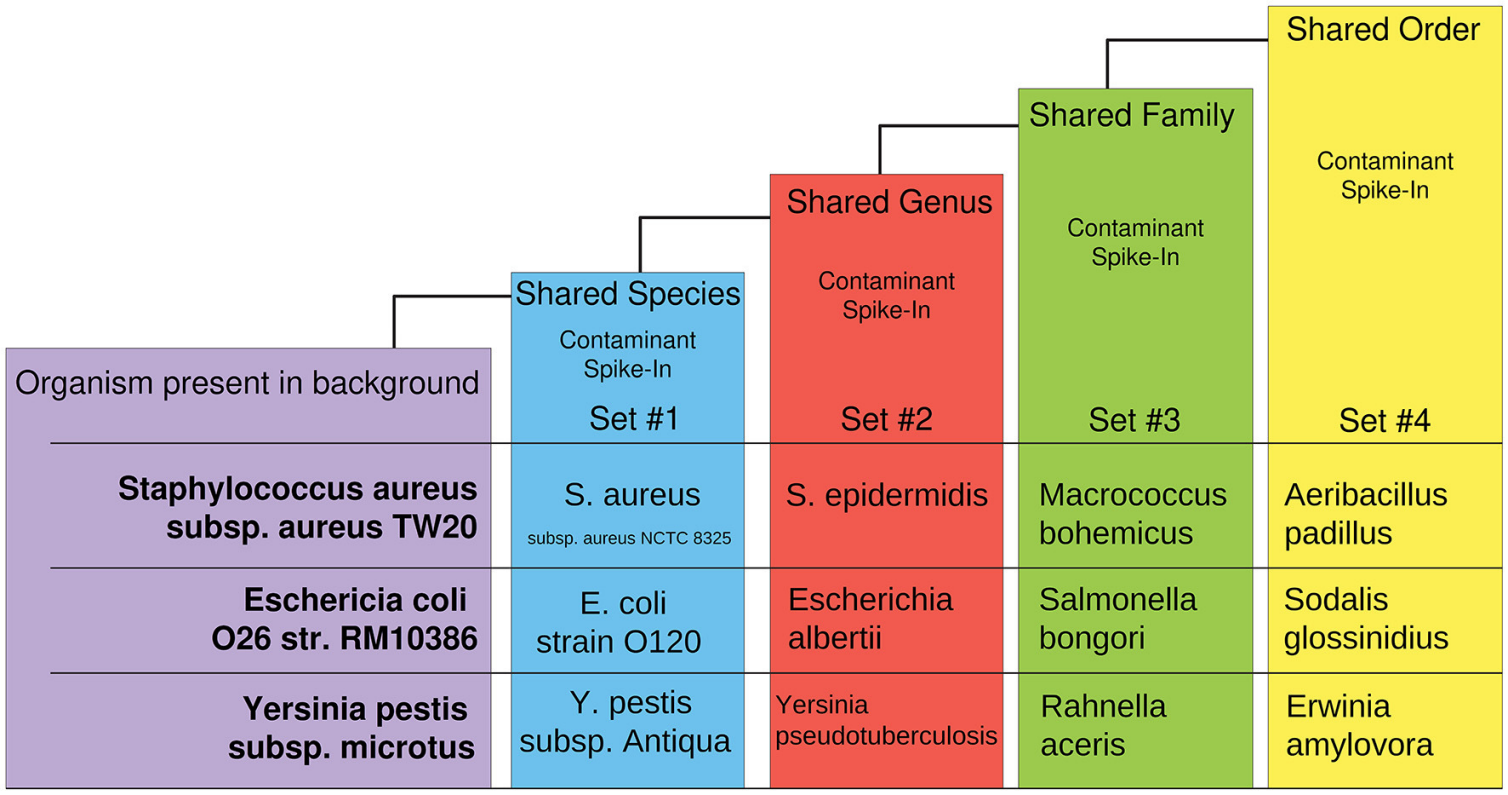
Overview of taxon-replacement strategy in CAMISIM-generated metagenomics anomaly datasets. Selected taxa within the “normal” conditions had increasingly distant taxonomic relatives substituted into “anomalous” metagenome profiles. Three different taxon-substitute datasets were constructed at four increasingly distant taxonomic levels from “normal” conditions.

The synthetic dataset was generated by CAMISIM using CAMISIM's *de novo* metagenomic simulation. In total, 50 organisms with numerous high-quality reference genomes were chosen to form a typical control organism background, and *n* = 100 *de novo* metagenomic samples were simulated. Additionally, 12 experimental “spike-in” datasets were created by including one additional taxon to the control set during *de novo* metagenome simulation and *n* = 50 metagenomic samples were simulated as a test set of anomalies for each. The 12 additional organisms were chosen at increasing levels of taxonomic distance (i.e., shared species, genus, family, or order) to an organism present in the non-anomalous “background” control (Figure 2). These datasets were designed to benchmark AMAnD's ability to discern taxonomy anomalies originating from very small taxonomic distances to increasingly large disparities and which modeling methods—PanGIA taxonomy profiles or k-mers—were more sensitive to these changes.

The CAMISIM experiments were replicated three times at different metagenome sample sizes to profile AMAnDs' performance as coverage varies. The simulation size parameter per dataset ranged across 0.1, 0.05, and 0.01 gigabase pairs. Individual spike-ins of anomalous organism sequence reads from the CAMISIM dataset test AMAnD in cases where a single taxon is anomalous. The Feehan et al. and Tian et al. datasets capture larger community abundance shifts in taxa that are already present. This range of testing allows a more complete picture of possible anomalies that would be desirable to detect in an operational setting.

The AMAnD code repository and documentation are available in full on GitHub at https://github.com/colinwprice/AMAnD under the open GLv3 license.

## 3. Results

### 3.1. CAMISIM dataset

**Figure 3:**
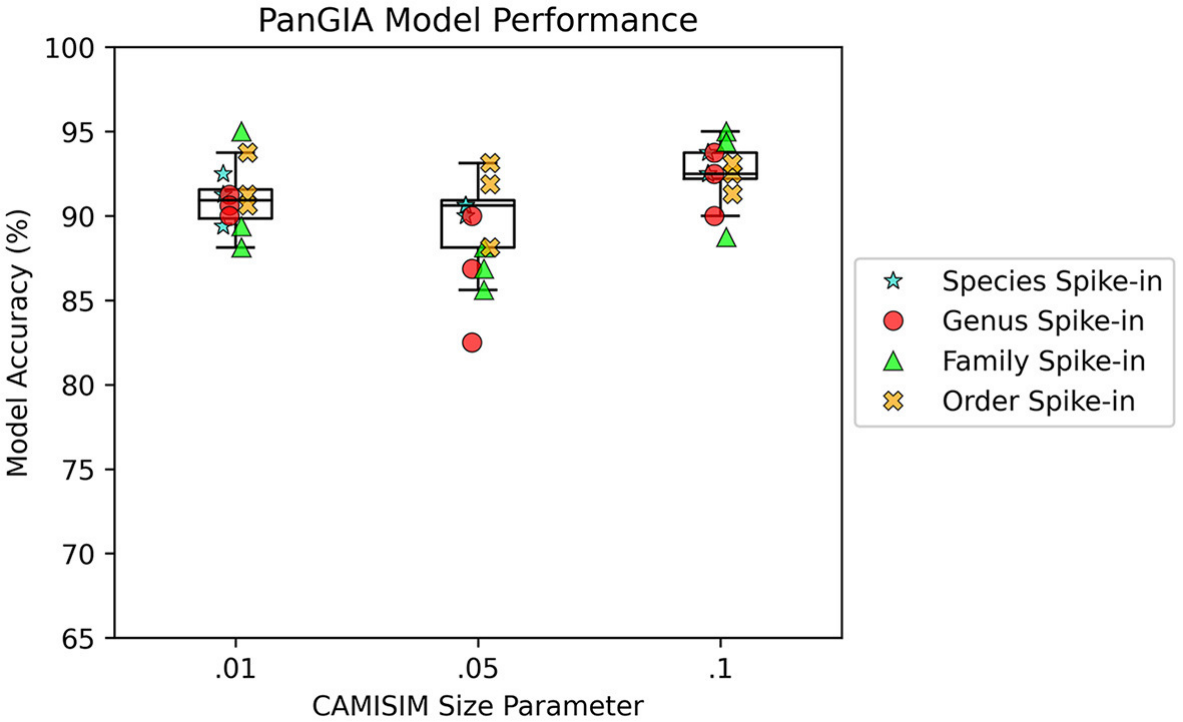
AMAnD's PanGIA model performance in microbial taxonomy feature space across different simulated coverage levels and increasing taxonomic distance for “anomalous” taxa.

**Figure 4:**
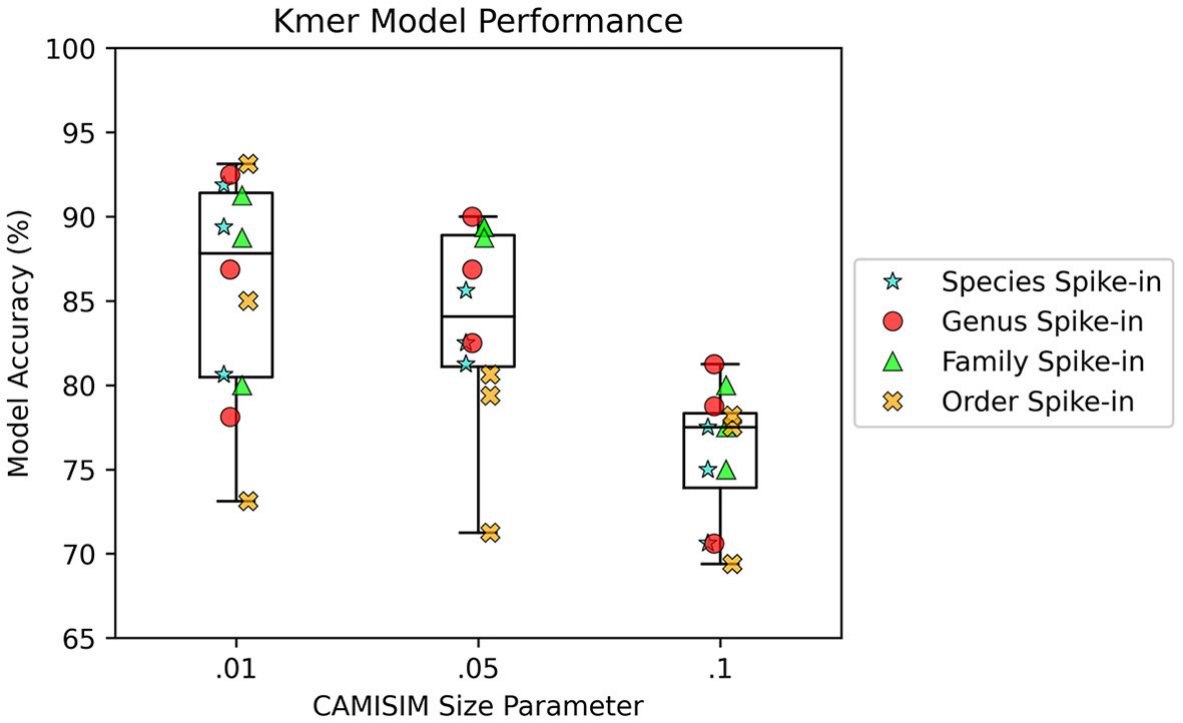
AMAnD's k-mer feature space model performance across different simulated coverage levels and increasing taxonomic distance for “anomalous” taxa.

AMAnD models trained on both k-mer feature vectors and PanGIA feature vectors were able to reliably differentiate the 12 spike-in anomaly cases from the “normal conditions” background cases. In the CAMISIM dataset experiments, the PanGIA models performed better. The highest accuracy across all optimized PanGIA feature vector models was achieved on the *Macrococcus bohemicus* spike-in condition at 95%, and the lowest accuracy was 85.63% on the *Rahnella aceris* spike-in condition (Figure 3; Supplementary Figure 4)—both of those anomaly targets were at shared family level taxonomic divergence. The mean performance of all optimized PanGIA feature vector models was at an accuracy of 91.15% (±2.21%), almost 10% better than the k-mer feature vector model average of 81.93% (±6.84%) which exhibited higher variance in performance across conditions (Figures 3, 4). Across all optimized k-mer feature vector models, the highest performance was the *Sodalis glossinidius* condition at 93.13% accuracy and the lowest was the *Erwinia amylovora* spike-in at 69.4% accuracy (Figure 4; Supplementary Figure 4)—interestingly, both of those anomaly targets were also at the shared family level taxonomic divergence. These results suggest that in cases where there is a single anomalous taxon present, regardless of the taxonomic similarity of that anomalous taxon to the normal conditions, the PanGIA feature vector is a more consistent choice with lower variance in the ability to correctly label anomaly from control. However, there are some specific conditions that the k-mer feature vector outperforms the PanGIA feature vector which suggests that considering an ensemble of both models would improve fidelity. For example, the *Escherichia albertii* condition at CAMISIM coverage simulation 0.01 has a higher k-mer model accuracy than the PanGIA counterpart (Supplementary Figure 4). Examining performance across the tiers of taxonomic similarity between “normal” background control and anomalous organism spike-ins, the average performance of PanGIA remained consistent, achieving average accuracies of 91.45, 91.25, 90.14, and 91.74% for anomaly targets diverging by strain, species, genus, and family levels, respectively (Figure 3; Supplementary Figure 4). The k-mer feature vector models exhibited more variance and appeared to improve as the spike-in organisms became more dissimilar from their counterparts in the “normal” background control, until the family level when performance dropped to the lowest value. Average accuracies were found to be 81.60, 83.05, 84.44, and 78.62% for anomaly targets diverging by strain, species, genus, and family levels, respectively (Figure 4; Supplementary Figure 4). When considering the data across both PanGIA and k-mer feature vectors, AMAnD's performance appears independent of taxonomy which is contrary to our initial hypothesis that performance would improve with increasing taxonomic divergence for anomaly organisms. The final consideration in the CAMISIM experimental cases is the simulation size for the metagenomic samples. The CAMISIM size parameter was set at 0.01, 0.05, and 0.1 gigabase pairs to investigate if performance would be impacted by the sequence read coverage across taxa in the metagenomic samples. The distributions and sample numbers were held constant between the different-sized simulations. In PanGIA feature vector models, performance was seemingly invariant to the depth-of-coverage of the metagenomic samples averaging 91.09% (±1.92%), 89.84% (±2.21%), and 92.50% (±1.78%) for the 0.01, 0.05, and 0.1 sizes, respectively (Figure 3). In k-mer feature vector models, performance was superior on lower coverage metagenomic samples with average accuracies found to be 85.89% (±6.52%), 83.95% (±5.52%), and 75.94% (±3.89%) for the 0.01, 0.05, and 0.1 sizes, respectively (Figure 4). Surprisingly, we find that there does not seem to be a pattern where more dissimilar spike-in organisms at the higher taxonomic branches like order perform better than strain difference spike-ins. We expect this is due to the representation between the anomalous and normal control being sufficiently different when a whole new organism is inserted into the sample at the same rate as the other 50 normal control organisms for both feature vectors.

### 3.2. Nasopharyngeal dataset

**Figure 5:**
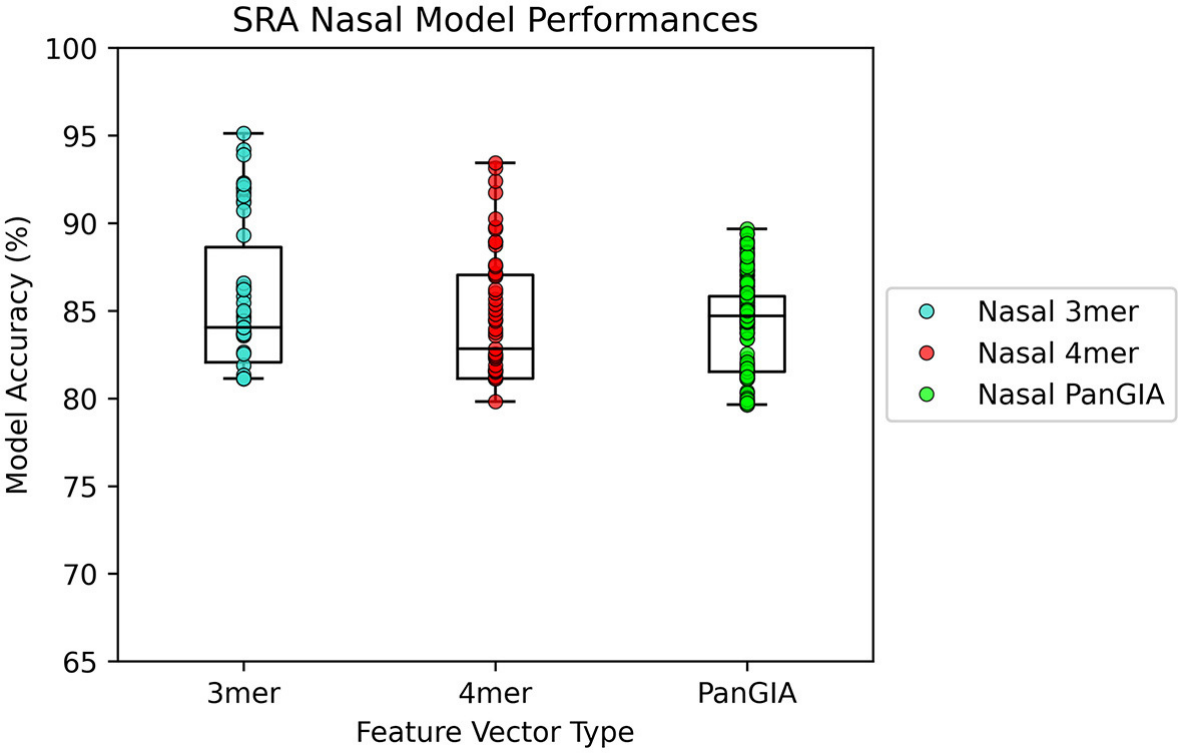
AMAnD's model performance distribution across all hyperparameter combinations evaluated for the NPS test sets.

**Figure 6:**
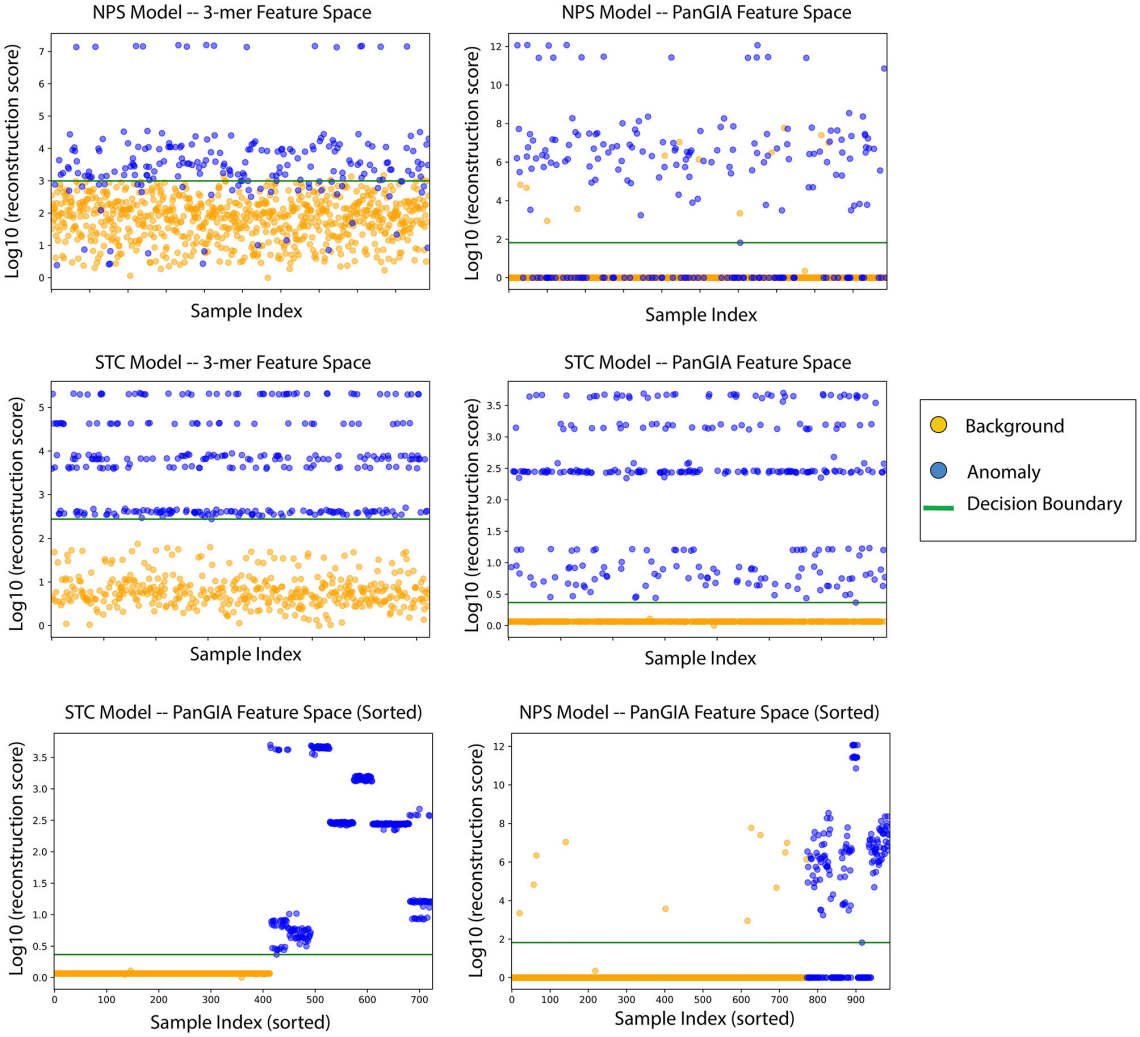
AMAnD's reconstruction score for training samples across k-mer and taxonomic feature spaces on NPS (top two plots) and STC conditions (middle two plots). When sorted by sample index number, the varied ways in which samples can present as anomalous are evidenced by the clustered groupings—AMAnD can accurately divide “normal” from “anomalous” despite this variation.

In the nasopharyngeal dataset retrieved from Feehan et al., both the k-mer feature vector and PanGIA feature vector were found to successfully differentiate the COVID-positive anomalous samples from the COVID-negative control samples. The optimal k-mer feature vector model was found to be 95.1% accurate, and the optimal PanGIA feature vector model was found to be 91% accurate (Figures 5, 6). The higher performance of the k-mer feature vector here may suggest that the PanGIA feature truncation approach used by AMAnD is less suited for highly diverse and varied metagenomic samples like those found in human microbiomes when compared to k-mers. Feehan et al. found significant differences in several taxonomic groups in COVID-positive patients which AMAnD can reject after learning the typical COVID-negative background.

### 3.3. Gut microbiome datasets

**Figure 7:**
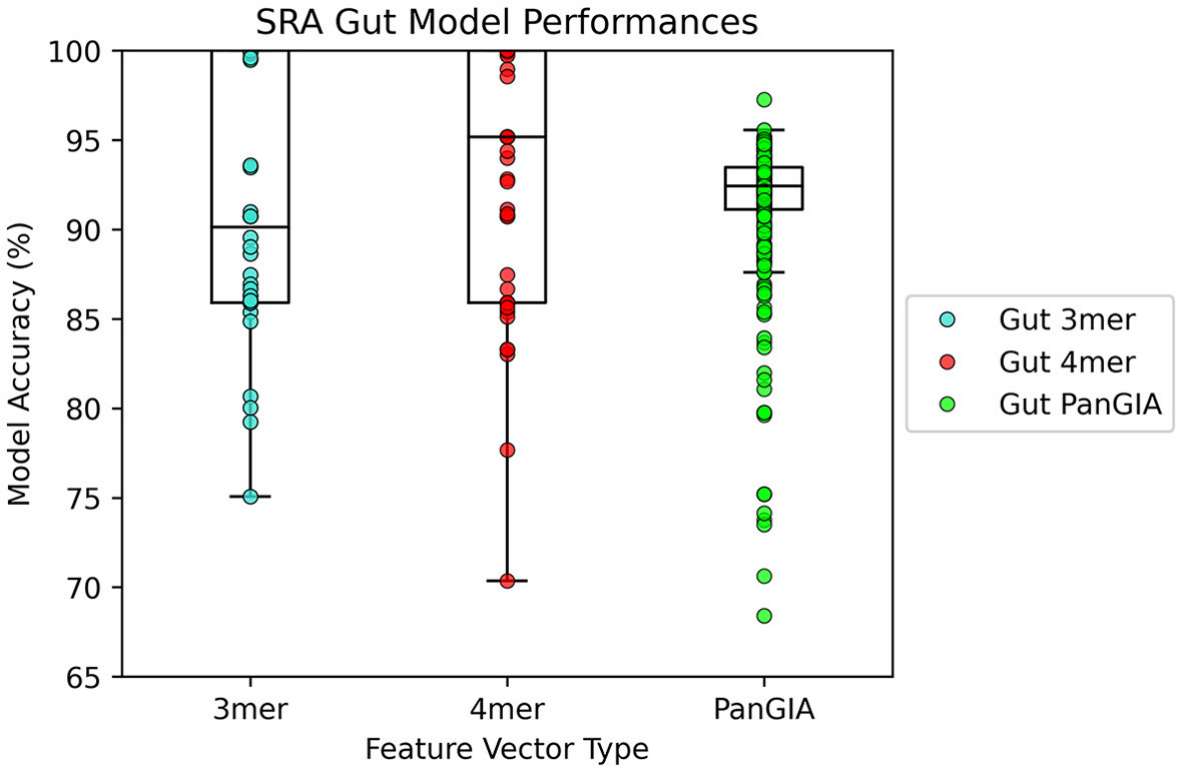
AMAnD's model performance distribution across all hyperparameter combinations evaluated for the STC test sets.

In the gut microbiome dataset retrieved from Tian's upload to SRA, both the k-mer feature vector and PanGIA feature vector again were found to successfully differentiate the anomalous slow transit constipation samples from the healthy control samples. The optimal k-mer feature vector model had 100% accuracy and the optimal PanGIA feature vector model had 97.3% accuracy (Figures 6, 7). This again mirrors the observations from the nasopharyngeal dataset, where the k-mer model outperforms the PanGIA model. Again, Tian's study found significant widespread changes in diversity distributions and compositions across many taxons at different taxonomic levels. The exhibited higher variance in the scoring of STC samples compared to the NPS is likely due to the higher species variance in the human gut microbiome.

When ordering the chunked metagenomic samples from the SRA studies, interesting patterns emerge that serve to validate the idea that AMAnD makes no assumptions about what an anomaly might look like *a priori*. That is, despite the relatively high diversity of a human microbiome sampled in a nasopharyngeal swab or the gut community, a consistent background is learnable. The anomaly samples, while all anomalous, are also anomalous in different ways as they have different error rates/scoring used in the classification. This becomes clearer when the chunked samples are ordered by their patient of origin, as despite COVID-positive patients and STC patients having the same condition within their anomaly group, it manifests in slightly different ways that are still flaggable by AMAnD (Figure 7).

Based on these results, we recommend that when deploying AMAnD to a regular sequencing operation both feature spaces that should be trained and considered. In the case where both models do not flag the sample, the sample is likely typical. In the case where either feature space flags the sample as anomalous, or if both feature space models flag the sample as anomalous, the sample should be further scrutinized for what the specific cause of the anomaly might be. AMAnD models will likely improve over time as the corpus of training data grows as well as with future additions of new feature spaces to consider side-by-side with current kmer and PanGIA feature spaces. Even though our findings are that in different metagenomic anomaly detection scenarios, one feature vector performs better, consideration of multiple feature vectors that perform well further augments AMAnD's strength of being assumption free; the more feature spaces represent the data the more likely it is to be representing the feature space that principally describes the potential anomaly.

## 4. Discussion

AMAnD demonstrates that a deep learning approach is well suited for metagenomic anomaly detection even at sample sizes as shown in the CAMISIM examples with a training size as small as *n* = 100. Intelligent curation of features descriptive of the metagenomic sample like PanGIA taxonomic read labelings or small k-mer abundances allows for small deep networks like AMAnD to have high fidelity. Compared to linear machine learning models like support vector machines or logistic regression models, neural networks like AMAnD have the advantage that they are better suited to “on-line training” deployment environments where individual samples can be passed as a one-off training instance to continuously update the weights of the network as more samples are validated as anomaly free, further improving the representation of typical background control learned by AMAnD. This is unlike support vector machines and other traditional machine learning methods that require the whole batch plus the one new sample to be passed through to retrain. In a production sequencing/regular sequencing environment, the option to immediately use the new data point and just the new data point each time is passed through the anomaly detector which is desirable to avoid long and less computationally efficient model retraining. DeepSVDD has been shown to hold an advantage over purely traditional auto-encoders with the additional fitting of a minimally sized n-dimensional hyper-sphere around the trained on normal control class.

Different AMAnD models are more effective at the different experimental anomaly sets. K-mer feature vector models likely performed better at low coverage due to the presence of the anomalous spike-in organism having a larger impact on the low k-mer distribution. In the PanGIA feature vector space, performance remained stable perhaps due to the fact that the relative abundance of the taxonomic spike-in organism with respect to the rest of the sample did not change.

AMAnD makes no assumptions about how an anomaly may be characterized, which makes it an appealing candidate for applications where there is little known about what constitutes a metagenomic anomaly. There is no information about the effects of prolonged deep spaceflight on the human metagenome (or the associated built environment of the crew cabin), so monitoring these samples for anomalous profiles should make as few assumptions as possible. While only a few types of anomalies are characterized here, if an anomaly would be represented in one or more feature vectors present in an ensemble of AMAnD models, AMAnD should be able to learn and flag it reliably. Furthermore, the small size of AMAnD's neural networks and the high fidelity of AMAnD on low metagenomic coverage make it attractive for space flight applications where lightweight and low-power solutions are desired, or other deployed operational environments where high-coverage data are challenging to acquire.

In practical deployment, models will likely not be selected based in part on test set performance as well as validation performance since a user will likely only have access to their “normal” class representatives to use as the only validation set for their hyperparameter tuning. It would be difficult to expect a user to come to the table with a handful of anomaly class representatives ahead of time, especially since the most valuable utility of this approach would mean there are no *a priori* assumptions on what an anomaly looks like. The test set here can be thought of almost as a second validation set for the model parameterizations; unlike a traditional mixed-class classification problem, only the normal control class is present in the validation and training data, meaning the test set characterizes the performance of rejecting the anomaly class while the minimum validation error characterizes the generalization to unseen control data that should not be rejected as anomalous. No model parameterization considered in this study should be considered as an “off the shelf” ready model but should instead show that diverse hyperparameterizations are viable on both validation and test datasets.

For future work on AMAnD, additional feature domains will be profiled to further validate the generalizable nature of AMAnD in areas such as protein space and gene space. Additionally, training an assembling meta-model to consolidate the voting of all feature spaces may lead to a superior fidelity of anomaly detection. A second validation stage could be added in which previously rejected anomalies are kept as a “second validation” set to re-validate the model each time the weights are updated over the lifetime of the model's deployment—this should ensure that labeling is still consistent with the previous iterations similar to how the test set was employed in model selection in this study. The collection of additional datasets along a temporal axis to profile how the performance of AMAnD models changes as additional data are trained over time would help uncover how the performance of AMAnD scales to high sample counts. Furthermore, the results of temporal metagenomic data analysis could provide interesting insight into how a metagenomic sequencing operation changes over time.

AMAnD's success with real-world metagenomic data that reflect different human pathologies suggests it is well-suited for applied metagenomics biosurveillance. The feature vectors of both k-mer frequency and PanGIA taxonomy demonstrated success on all benchmarked datasets and show potential to be used in an ensemble to further bolster robustness to different anomaly types. PanGIA feature vectors capture small changes in taxonomic composition and k-mer frequency can capture non-taxonomic shifts in microbial communities. AMAnD models could be trained on a variety of other possible metagenomic descriptive feature vectors, including gene annotations, degenerate k-mer vectors (26), amino acid k-mers, and nanopore electrical signals (27–29). Aggregating many AMAnD models together across different feature vector types will further improve the generalizability of anomaly detection.

## Data availability statement

Publicly available datasets were analyzed in this study. This data can be found here: Nasopharyngeal Microbiome Community Composition and Structure is Associated with Severity of COVID-19 Disease and Breathing Treatment (Feehan), https://www.ncbi.nlm.nih.gov/sra, SRR15046431–SRR15046439, SRR15046448, SRR15046450–SRR15046476; Analysis of Gut Microbiome and Metabolite Characteristics in Patients with Slow Transit Constipation (Tian), https://www.ncbi.nlm.nih.gov/sra, SRR16961778–SRR16961827; Dryad dataset hosting of genomes used by CAMISIM simulation (has associated https://doi.org/10.5061/dryad.t4b8gtj68): https://datadryad.org/stash/share/uJDp699QyHQw6FnLcdsNvsNtG1P8pJkJADgxTsCcwOc.

## Author contributions

Work was conceptualized by CP and JR. Code was developed by CP. Data was analyzed by CP and JR. Manuscript was written by CP and JR. All authors contributed to the article and approved the submitted version.

## References

[1] Bonomo MG Calabrone L Scrano L Bufo SA Di Tomaso K Buongarzone E. Metagenomic monitoring of soil bacterial community after the construction of a crude oil flowline. Environ Monit Assess. (2022) 194:48. 10.1007/s10661-021-09637-3 34978609 PMC8724107

[2] Brumfield KD Leddy M Usmani M Cotruvo JA Tien CT Dorsey S. Microbiome analysis for wastewater surveillance during COVID-19. MBio. (2022) 13:e00591–22. 10.1128/mbio.00591-22 35726918 PMC9426581

[3] Lee MD O'Rourke A Lorenzi H Bebout BM Dupont CL Everroad RC. Reference-guided metagenomics reveals genome-level evidence of potential microbial transmission from the ISS environment to an astronaut's microbiome. Iscience. (2021) 24:102114. 10.1016/j.isci.2021.102114 33659879 PMC7892915

[4] Parker K Wood H Russell JA Yarmosh D Shteyman A Bagnoli J. Development and optimization of an unbiased, metagenomics-based pathogen detection workflow for infectious disease and biosurveillance applications. Trop Med Infect Dis. (2023) 8:121. 10.3390/tropicalmed8020121 36828537 PMC9966482

[5] Chiu CY Miller SA. Clinical metagenomics. Nat Rev Genet. (2019) 20:341–55. 10.1038/s41576-019-0113-7 30918369 PMC6858796

[6] Thomas MB. Epidemics on the move: climate change and infectious disease. PLoS Biol. (2020) 18:e3001013. 10.1371/journal.pbio.3001013 33232329 PMC7685491

[7] Gómez-Tatay L Hernández-Andreu JM. Biosafety and biosecurity in synthetic biology: a review. Crit Rev Environ Sci Technol. (2019) 49:1587–621. 10.1080/10643389.2019.1579628

[8] Breitwieser FP Lu J Salzberg SL. A review of methods and databases for metagenomic classification and assembly. Brief Bioinform. (2019) 20:1125–36. 10.1093/bib/bbx120 29028872 PMC6781581

[9] Eisenhofer R Minich JJ Marotz C Cooper A Knight R Weyrich LS. Contamination in low microbial biomass microbiome studies: issues and recommendations. Trends Microbiol. (2019) 27:105–17. 10.1016/j.tim.2018.11.003 30497919

[10] Jansson JK Hofmockel KS. Soil microbiomes and climate change. Nat Rev Microbiol. (2020) 18:35–46. 10.1038/s41579-019-0265-7 31586158

[11] Feehan AK Rose R Nolan DJ Spitz AM Graubics K Colwell RR. Nasopharyngeal microbiome community composition and structure is associated with severity of COVID-19 disease and breathing treatment. Appl Microbiol. (2021) 1:177–88. 10.3390/applmicrobiol1020014

[12] Dubinkina VB Ischenko DS Ulyantsev VI Tyakht AV Alexeev DG. Assessment of k-mer spectrum applicability for metagenomic dissimilarity analysis. BMC Bioinformatics. (2016) 17:1–11. 10.1186/s12859-015-0875-7 26774270 PMC4715287

[13] Ghannam RB Techtmann SM. Machine learning applications in microbial ecology, human microbiome studies, and environmental monitoring. Comput Struct Biotechnol J. (2021) 19:1092–107. 10.1016/j.csbj.2021.01.028 33680353 PMC7892807

[14] Ren J Song K Deng C Ahlgren NA Fuhrman JA Li Y. Identifying viruses from metagenomic data using deep learning. Quant Biol. (2020) 8:64–77. 10.1007/s40484-019-0187-4 34084563 PMC8172088

[15] Liu Y Elworth RL Jochum MD Aagaard KM Treangen TJ. De novo identification of microbial contaminants in low microbial biomass microbiomes with Squeegee. Nat Commun. (2022) 13:6799. 10.1038/s41467-022-34409-z 36357382 PMC9649624

[16] Khan SS Madden MG. A survey of recent trends in one class classification. In: Artificial Intelligence and Cognitive Science: 20th Irish Conference, AICS 2009, Dublin, Ireland, August 19-21, 2009, Revised Selected Papers 20 (Springer Berlin Heidelberg) (2010). p. 188–97.

[17] Zhou C Paffenroth RC. August. Anomaly detection with robust deep autoencoders. In: Proceedings of the 23rd ACM SIGKDD International Conference on Knowledge Discovery and Data Mining. (2017). p. 665–74.

[18] An J Cho S. Variational autoencoder based anomaly detection using reconstruction probability. Special Lecture IE. (2015) 2:1–18.

[19] Ruff L Vandermeulen R Goernitz N Deecke L Siddiqui SA Binder A. Deep one-class classification. In: International Conference on Machine Learning. PMLR. (2018). p. 4393–402.

[20] Zhang Z Deng X. Anomaly detection using improved deep SVDD model with data structure preservation. Pattern Recognit Lett. (2021) 148:1–6. 10.1016/j.patrec.2021.04.020

[21] Han S Hu X Huang H Jiang M Zhao Y. Adbench: anomaly detection benchmark. arXiv preprint. (2022). 10.2139/ssrn.4266498

[22] Li E Russell JA Yarmosh D Shteyman AG Parker K Wood H. PanGIA: a metagenomics analytical framework for routine biosurveillance and clinical pathogen detection. bioRxiv. (2020). 10.1101/2020.04.20.051813

[23] Marcais G Kingsford C. A fast, lock-free approach for efficient parallel counting of occurrences of k-mers. Bioinformatics. (2011) 27:764–70. 10.1093/bioinformatics/btr011 21217122 PMC3051319

[24] Fritz A Hofmann Majda S Dahms E Dröge J Fiedler J. CAMISIM: simulating metagenomes and microbial communities. Microbiome. (2019) 7:1–12. 10.1186/s40168-019-0633-6 30736849 PMC6368784

[25] Tian H Chen Q Yang B Qin H Li N. Analysis of gut microbiome and metabolite characteristics in patients with slow transit constipation. Dig Dis Sci. (2021) 66:3026–35. 10.1007/s10620-020-06500-2 32767153

[26] Davis P Russell JA. A genotype-to-phenotype modeling framework to predict human pathogenicity of novel coronaviruses. bioRxiv. (2021). 10.1101/2021.09.18.460926

[27] Loose M Malla S Stout M. Real-time selective sequencing using nanopore technology. Nat Methods. (2016) 13:751–4. 10.1038/nmeth.3930 27454285 PMC5008457

[28] Bao Y Wadden J Erb-Downward JR Ranjan Zhou W McDonald TL. SquiggleNet: real-time, di-rect classification of nanopore signals. Genome Biol. (2021) 22:1–16. 10.1186/s13059-021-02511-y 34706748 PMC8548853

[29] Payne A Holmes N Clarke T Munro R Debebe BJ Loose M. Readfish enables targeted nanopore se-quencing of gigabase-sized genomes. Nat Biotechnol. (2021) 39:442–50. 10.1038/s41587-020-00746-x 33257864 PMC7610616

