# Supplementary material for: AMAnD: an automated metagenome anomaly detection methodology utilizing DeepSVDD neural networks
Source: Front Public Health. 2023 Jul 11;11:1181911. doi: 10.3389/fpubh.2023.1181911 (PMC10368493; doi:10.3389/fpubh.2023.1181911)
Supplement: Supplementary file 1 [file Table_1.DOCX]

Supplementary Material

AMAnD: an Automated Metagenome Anomaly Detection methodology utilizing DeepSVDD neural networks

**Colin Price**^1^, **Joseph A. Russell^1^**

*** Correspondence:** Colin Price: cprice@mriglobal.org

## Supplementary Figures


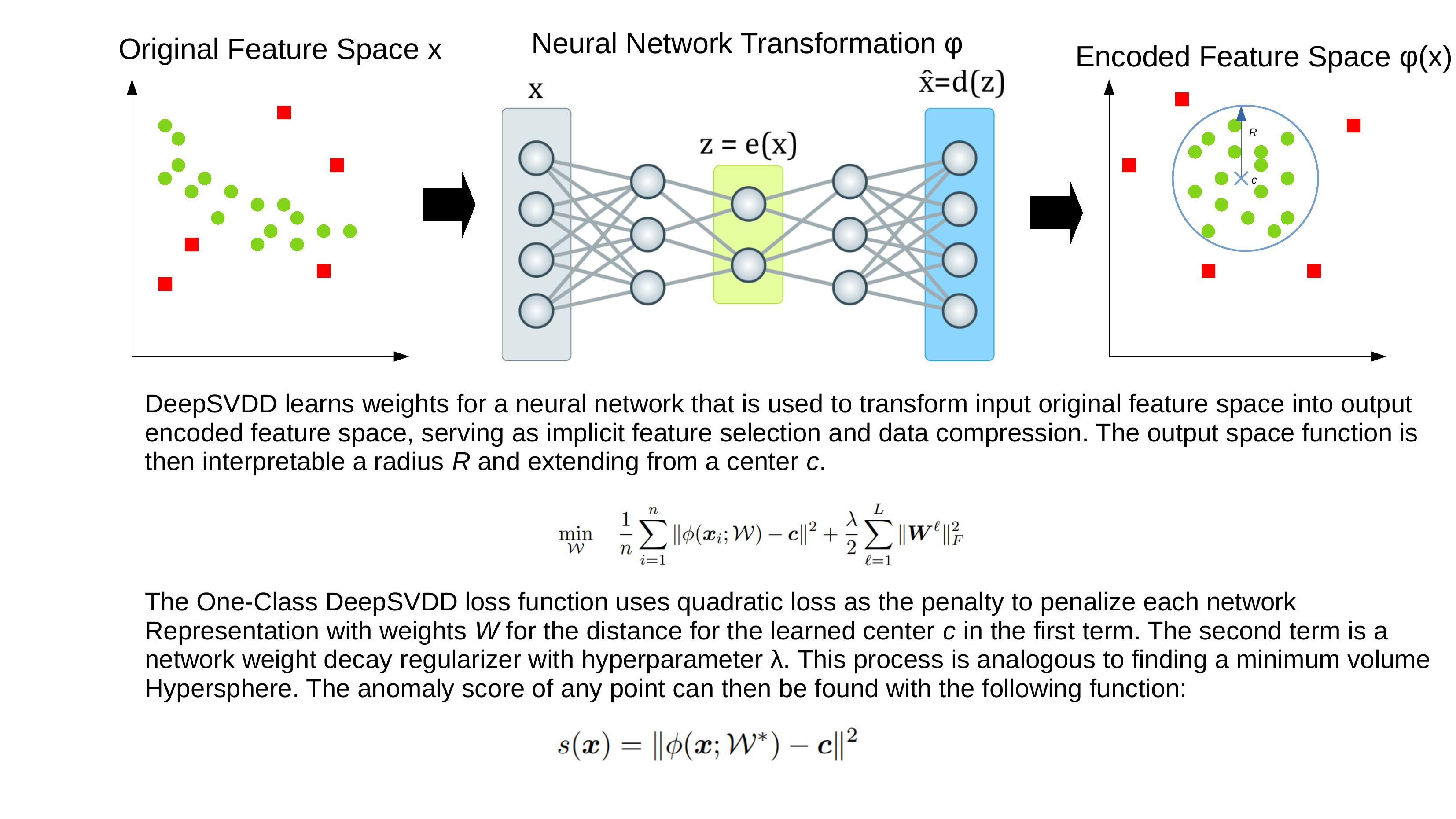
**Supplementary Figure 1**

Details on the objective function and core principals behind the DeepSVDD model employed by AMAnD.


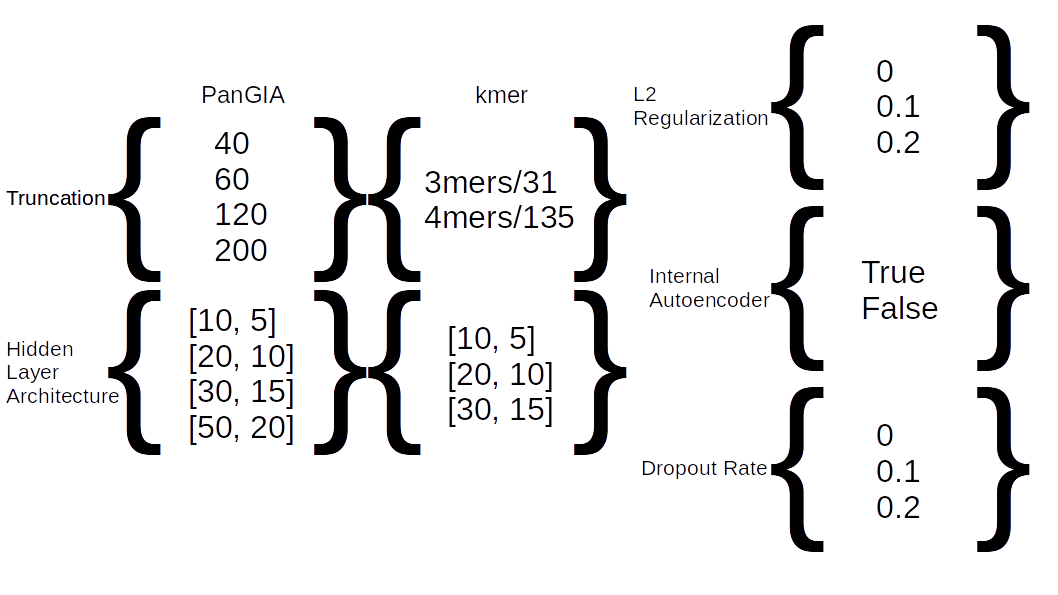
**Supplementary Figure 2**

Parameterizations of the DeepSVDD module in PyOD’s library. Hidden layer architecture represents the number of nodes on the layer in the format [outer, inner]. Kmer truncations are constrained to the sizes of canonical kmer representations.

**

**

**Supplementary Figure 3**

Selected training profiles for 5 organisms (A. pallidus, M. bohemicus, S. bongori, S. aureus, and Y. pestis) across both kmer and PanGIA classification feature spaces and across increasing dataset coverage levels (0.01 Gb, 0.05 Gb, and 0.10 Gb). Training and validation scores converge at some level across all examples. Importantly, when one feature space does not converge as well, the accompanying feature space exhibits a better profile, underscoring the robustness of the binary-feature approach.


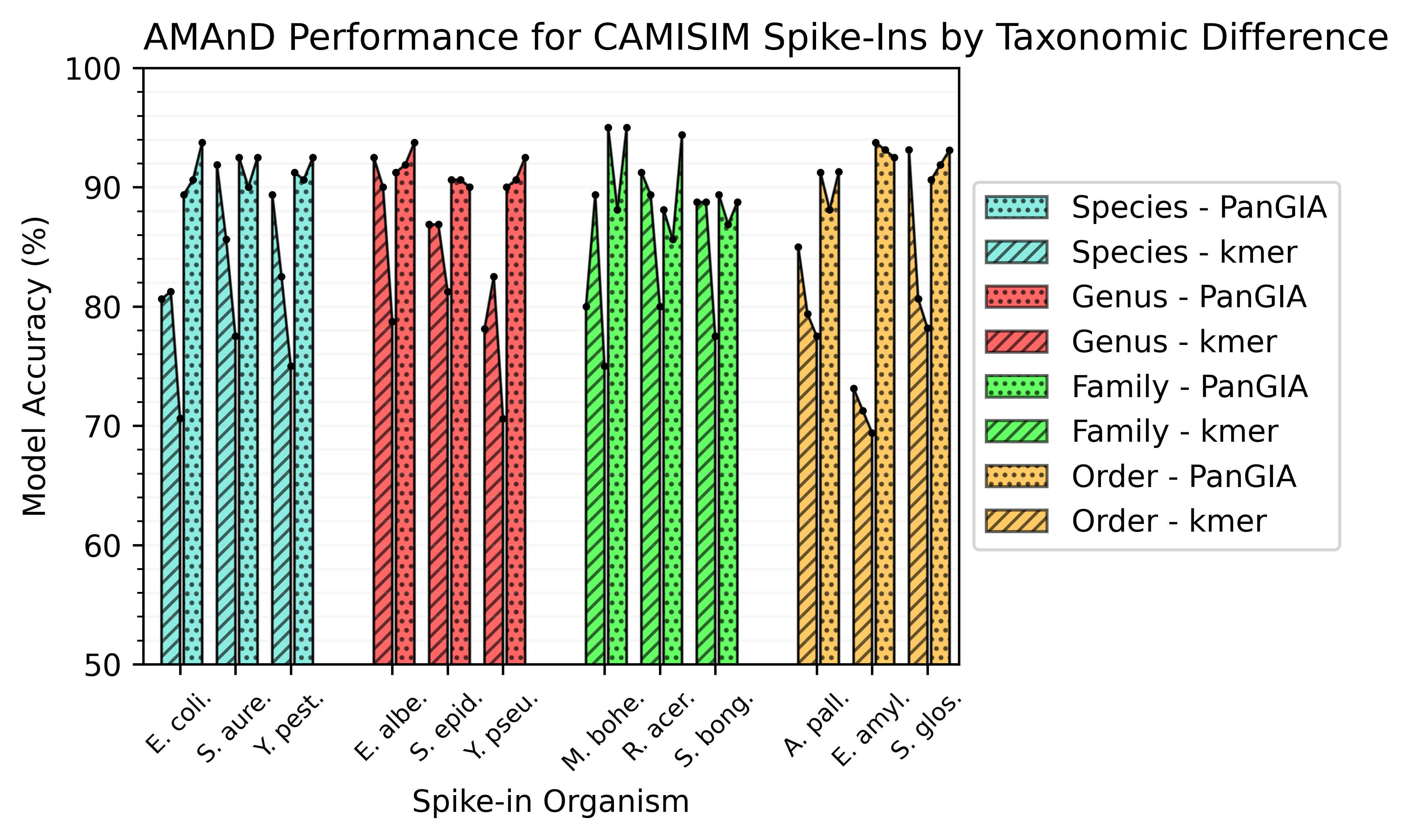
**Supplementary Figure 4**

Plot of AMAnD model performance for all AMAnD models, separated by taxonomic difference, feature vector type (PanGIA or kmer), and size (first point on bar plot is size=.01, middle is size=.05, last is size=.1)

**1.2 Supplementary Tables**


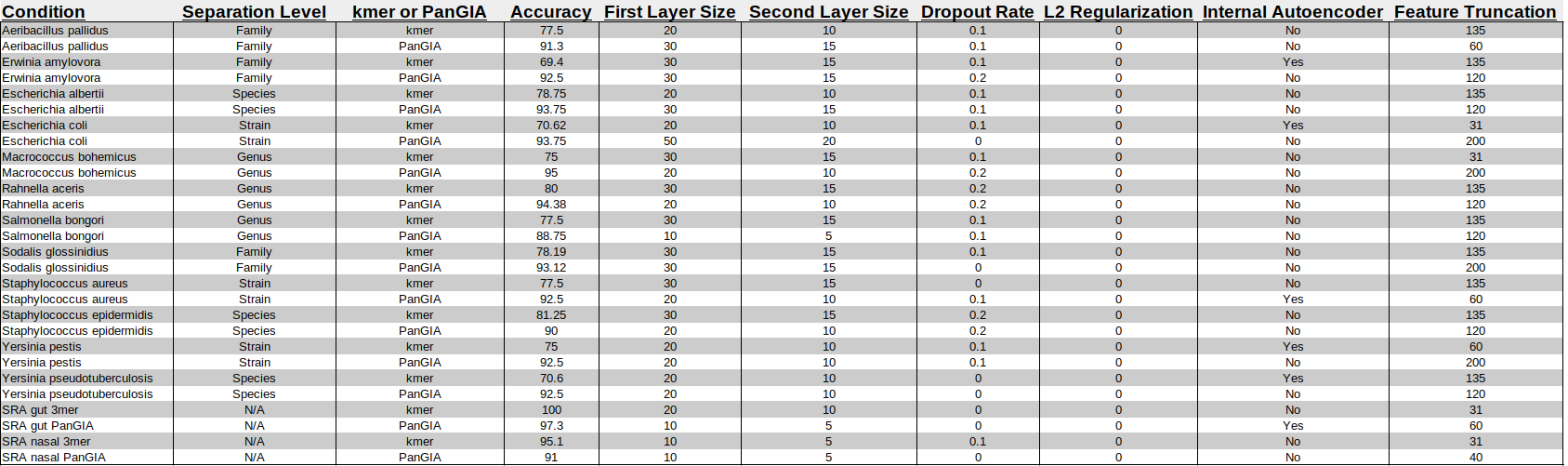
**1.3 Supplementary Code**

The AMAnD code repository and documentation is available in full on Github at <https://github.com/colinwprice/AMAnD> under the open GLv3 license.
